# Between the Trees: Quantifying Koala Ground Movement for Conservation Action

**DOI:** 10.3390/ani15243537

**Published:** 2025-12-08

**Authors:** Gabriella R. Sparkes, Oakleigh Wilson, William A. Ellis, Sean I. FitzGibbon, Benjamin J. Barth, Christofer J. Clemente, Mathew S. Crowther, Robbie S. Wilson

**Affiliations:** 1 School of the Environment, University of Queensland, Brisbane, QLD 4072, Australia; g.sparkes@uq.edu.au (G.R.S.); w.ellis@uq.edu.au (W.A.E.);; 2 School of Science, Technology and Engineering, University of the Sunshine Coast, Sippy Downs, QLD 4556, Australia; oakleigh.wilson05@gmail.com (O.W.); cclement@usc.edu.au (C.J.C.); 3 School of Life and Environmental Sciences, University of Sydney, Sydney, NSW 2006, Australia; mathew.crowther@sydney.edu.au

**Keywords:** accelerometry, machine learning, movement ecology, koala conservation, behavioural ecology

## Abstract

Koalas are an iconic Australian species now classified as nationally endangered due to habitat loss, predation, disease, and vehicle collisions. Yet, because of their nocturnal and arboreal nature, koala movement on the ground between trees remains poorly described. Ground visits are fraught with danger, with two-thirds of koala deaths occurring due to vehicle strikes and dog attacks. Quantifying these behaviours can provide insights for conservation efforts targeted to their time on the ground. To quantify detailed movement patterns, we collared nine koalas in a highly fragmented agricultural landscape with tri-axial accelerometers for 8.50 ± 1.17 days. Using an annotated dataset of observed behaviours, we trained Random Forest models to classify four main behavioural states: *Motionless in Tree*, *Feeding & Grooming in Tree*, *Other Movement in Tree*, and *Walking*. Koalas spent 57.5% ± 14.2% of their time moving in trees, 26.8% ± 13.5% motionless in trees, 15.6% ± 2.7% feeding and grooming, and only 0.2% ± 0.1% walking on the ground (around 3 min daily). Koalas walked most frequently between 2 am and 5 am. Our findings highlight a major discrepancy between mortality rates on the ground and frequency of ground time, indicating that focused mitigation during these infrequent events could yield disproportionately high conservation benefits.

## Simple Summary

This study addresses the limited understanding of koala movement on the ground—a behaviour that, while representing only a small fraction of their day, poses the greatest risk to their survival. Koalas, now listed as nationally endangered, face major threats from habitat loss, dog attacks, and vehicle collisions, with most fatalities occurring during brief ground movements between trees. We quantify these ground-based behaviours and identify when koalas are most vulnerable. Nine wild koalas in a fragmented agricultural landscape were fitted with accelerometer collars for 8–10 days, and their movements were classified using machine learning models into four behavioural states. Koalas spent over half their time moving in trees, about a quarter resting, and less than 1% (around three minutes daily) walking on the ground, mostly between 2 am and 5 am. The stark mismatch between time spent on the ground and mortality risk reveals a critical conservation opportunity: targeted mitigation strategies—speed reductions, wildlife crossings, and dog management policies—during these narrow nocturnal windows can maximise conservation impact while minimising costs. This research provides new insight into fine-scale koala behaviour, informing more effective, evidence-based conservation strategies for the species.

## 1. Introduction

Koalas (*Phascolarctos cinereus*) spend most of their lives in the canopy, relying entirely on trees for food, shelter, and rest [1,2]. As specialist folivores, they feed almost exclusively on a narrow range of eucalypt species, with selection of fodder trees influenced by leaf chemistry and moisture content [3,4,5,6,7]. Koalas are largely sedentary, sleeping for up to 20 h per day and spending the remainder of their time feeding, grooming, or moving between trees [8,9]. Koala populations have declined by more than 50% in parts of eastern Australia over the past two decades alone [10,11], and the species is now listed as Endangered in Queensland, New South Wales, and the Australian Capital Territory under Australian Commonwealth legislation [12]. The major causes of mortality include disease (predominantly chlamydiosis, 34%), dog attacks (14%), and vehicle strikes (52%), all of which are exacerbated by habitat loss and land-use change [13,14,15,16]. In intact forests, koalas typically move between neighbouring trees using short ground movements, as trees are closer together [14]. However, in fragmented landscapes, koalas spend more time on the ground and travel greater distances to reach trees [17,18]. Although such movements are both essential and expose koalas to increased risks, including predation, vehicular collision, and livestock, they remain poorly described [19]. Understanding the patterns of movement by koalas when on the ground may allow us to identify specific periods and locations that are high-risk, supporting actions that could improve landscape connectivity and help reduce mortality from vehicles and dogs [20]. This information is critical for reducing preventable deaths and ensuring that fragmented habitats remain viable for koala populations.

In Queensland, koalas are primarily nocturnal, often selecting large non-fodder trees with dense canopies for daytime resting and thermoregulation, and seeking preferred food trees at night [21,22,23]. Thus, movements on the ground occur mostly at night, making these behaviours difficult to observe directly [18,24,25,26]. Studies using GPS and VHF collars suggest koalas spend 0.7–10% of their day on the ground, typically descending once or twice per night and travelling anywhere from a few metres to over 600 m in a single movement bout [24,27,28]. However, these estimates vary widely across populations, habitat types, and seasons, and methodological limits add uncertainty [29,30]. GPS fixes are often 10–15 min apart—or, more commonly, only once or twice per day—potentially overestimating short ground walks, while canopy cover can reduce location accuracy, add noise, and result in data loss, ultimately making it difficult to determine if the koala is in a tree or on the ground [31,32]. Many studies have therefore relied on direct observation to confirm ground use or road-crossings, but this is expensive, time-consuming, and can interfere with the behaviours being observed [33]. Acoustic monitoring has been used to detect koala behaviours with high temporal precision, but the manual analysis of recordings is prohibitively time-intensive, limiting sample sizes [34]. These challenges highlight the need for tools that record continuous high-frequency data and analytical approaches that can process large datasets and detect and quantify ground-use behaviours without biases from GPS error or observer presence.

Accelerometers can capture continuous data, allowing an interpretation of an animal’s behaviour and movement at fine temporal scales. Accelerometers can record acceleration across three axes (heave, surge, sway) at high frequencies (e.g., 10–100 Hz; [35,36]). As specific behaviours are often associated with movements that generate distinct acceleration patterns across the three axes, it is possible to identify and classify behaviours based on their unique acceleration signatures. For example, steady oscillations may indicate walking, while flat signals can indicate resting [37]. Behavioural classification typically involves observing animals while they are wearing accelerometers, and then matching each behaviour to its unique signal in acceleration across the three axes. Such behavioural patterns can then be used to train machine learning models to predict behaviours in unclassified data, often from wild animals [38]. This approach is especially valuable for cryptic or canopy-dwelling species that are challenging to observe [36]. For example, accelerometers have distinguished resting, feeding, climbing, and locomotion in arboreal animals such as slow lorises [39], possums [40], and gliders [41], differentiating canopy from ground activity. Other features extracted from acceleration data, such as amplitude, frequency, and overall or vectorial dynamic body acceleration (ODBA/VeDBA), have also been used to estimate activity-specific energy costs and quantify time–energy budgets across free-ranging species [35,42,43,44,45,46,47]. In koalas, ODBA has been used to separate broad ‘inactive’ (resting, sleeping) from ‘active’ (grooming, feeding, moving) states [9], but finer behaviours such as ground walking remain unquantified. High-resolution accelerometry combined with machine learning offers a way to capture and quantify these infrequent, cryptic events.

Here, we apply this approach to quantify high-risk ground-based movements—specifically, when, how often, and for how long koalas walk on the ground between trees. We captured ten koalas in a highly fragmented agricultural landscape in south-east Queensland and fitted each with a collar containing a GPS, VHF, and tri-axial accelerometer device. Using accelerometer data recorded at 50 Hz, we trained a supervised machine learning model on captive koalas and applied it to data obtained from free-roaming koalas to separate arboreal from ground movement and quantify walking events. We then described daily activity patterns, estimated the energetic costs of arboreal and ground behaviours, and compared walking behaviour between koalas in forest patches and narrow roadside vegetation strips. We predicted: (1) walking on the ground would be an infrequent but detectable behaviour, (2) walking would occur most frequently at night and during crepuscular periods, (3) walking would require more energy than most arboreal behaviours, and (4) koalas in linear vegetation patches would spend more time on the ground than those in forest patches. This fine-scale approach reveals the timing, frequency, and effort of ground travel relative to arboreal behaviours, offering new insights into one of the riskiest moments in a koala’s day—when they leave the safety of the canopy to navigate the danger zone below.

## 2. Materials and Methods

### 2.1. Study Area

**Figure 1:**
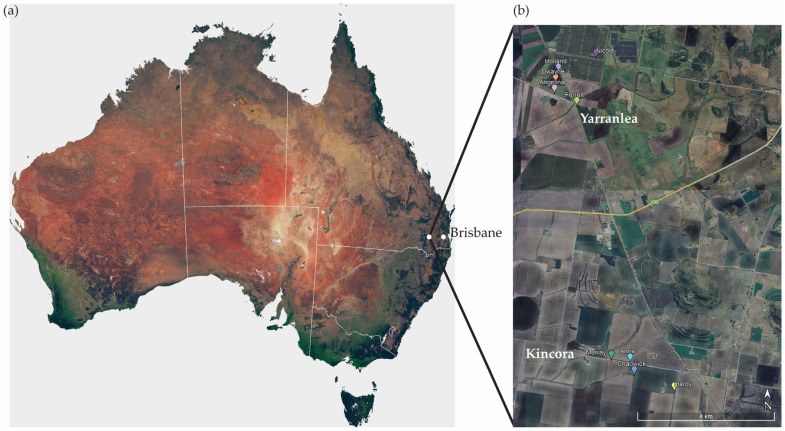
Map (Google Earth Pro, v7.3.6.10441) of (**a**) study site located in the Toowoomba region in south-east Queensland, and (**b**) specific locations in Yarranlea and Kincora of each koala tracked in the study at the initial capture point, individuals are identified by coloured pins (*n* = 6 males, 3 females).

Data were collected on wild koalas located around Kincora and Yarranlea in the Toowoomba Region, south-eastern Queensland (27°43′32.0” S, 151°31′46.5” E) over a three-week period in May–June 2023 (Figure 1). The landscape comprises a matrix of intensively farmed agricultural land interspersed with isolated open woodland patches and linear vegetation dominated by poplar box (*Eucalyptus populnea*), yellow box (*E. melliodora*), and scattered *Acacia* and *Casuarina* species. The study site is situated at 413–430 m elevation, has a temperate climate, and receives an average annual rainfall of 697 mm [48,49]. Mean daily temperatures for May–June range from 8.8 °C to 21.9 °C [48]. The region uses Australian Eastern Standard Time (UTC + 10) year-round, with no daylight savings adjustment. During the study period, civil dawn occurred at ~06:30 and dusk at ~17:15, which were used for activity analyses below.

### 2.2. Animals Used in the Study

We captured ten wild koalas (*P. cinereus*) using the ‘flag and pole’ technique [50]. Upon capture, each koala was fitted with a custom-built collar containing an accelerometer-gyroscope device (6-Axis Logging, AX6, Axivity, OpenMovement, Newcastle upon Tyne, UK), a GPS logger (igotU GT-600B, Mobile Action Technology, Inc., Taipei, Taiwan), and a VHF transmitter (A2650, Advanced Telemetry Solutions, Isanti, MN, USA), weighing no more than 115 g total (< 5% body mass). For each koala, we recorded sex, mass (kg), head width (mm), head length (mm), tooth wear (as an age proxy), and reproductive status. Mass was recorded using a digital hanging scale (15 kg model, WeiHeng WH-A10, Wuxi, China) minus the weight of the catch bag, and head measurements were recorded using Vernier callipers (±1 mm). Females were assessed for pouch young or signs of recent weaning (swollen teats), and males for sternal gland activity indicating reproductive maturity [51,52]. The average mass of males was 7.54 kg ± 0.57 kg (*n* = 6), and all had active sternal glands. The average mass of females was 5.80 kg ± 1.41 kg (*n* = 4), and they either had pouch young or had recently weaned. All koalas were classed as adults based on tooth wear and reproductive maturity. Once collared, koalas were released approximately 20 m from their capture tree to allow video recording of their movement during release, which was used in subsequent analyses of walking and ground-based movement. Koalas were then left unobserved for the duration of the three-week study period, by which time all device batteries were empty. At the end of this period, koalas were located using VHF and recaptured, after which we removed their collars and released koalas at their capture tree. One collar could not be retrieved as the female koala could not be caught safely. Thus, data from nine out of ten collars were used in the study, comprising 3 females and 6 males.

### 2.3. Device Orientation and Fixing Schedules

Accelerometers were positioned dorsally in a consistent orientation across individuals to allow comparative analyses. The AX6 device recorded tri-axial acceleration (heave, surge, sway) and tri-axial gyroscopic rotation (pitch, roll, yaw) at 50 Hz, ±4 G, and 250 dps. This configuration provided 50 data points per second per axis. Accelerometers recorded data for a mean of 8.50 ± 1.17 days. GPS devices recorded location every 5 min, with increased frequency (every 5 s) triggered if movement exceeded 1 km·h^−1^ to ensure all movement along the ground was captured. Logging paused if no movement was detected for more than 30 minutes to preserve battery life. GPS data were recorded for a mean of 11.97 ± 5.09 days. Data extracted from GPS devices were used in a subsequent study.

### 2.4. Collecting the Training Dataset

To train a supervised machine learning model for behaviour classification, we collected data from two captive koalas at Hidden Vale Research Station, Grandchester, Queensland (27°43′00.8″ S 152°27′47.3″ E), in November 2022. Koalas were housed in custom-built aviaries (5.6 mL, 3.6 mW, 3 mH) containing tree branches, branch forks, fresh foliage, and ground cover (e.g., rocks, logs, shrubs). Each koala was fitted with an identical collar to those used on wild koalas. Koalas were filmed continuously over a six-day period using a multi-camera setup: (1) four infrared CCTV cameras (HikVision HIK-2CD2142FWD14, Brisbane, Australia; two ground-level, two branch-height), (2) two top-down wide-angle fixed cameras, and (3) two wide-angle cameras (GoPro HERO8 Black, San Mateo, CA, USA). To ensure as many behaviours were captured in the training data as possible, we collected extra training data from two of the nine free-roaming koalas immediately after release to cover behaviours that were restricted in the captive setting, including longer bouts of climbing, walking, bounding, and branch-walking. Walking was the only behaviour for which we obtained enough samples from these additional data to use in the model.

### 2.5. Behavioural Recording and Classification

To synchronise accelerometer traces with behaviours observed on video, the collar was tapped five times in view of all cameras multiple times per day throughout the captive study period, creating a unique calibration timestamp. The accelerometer traces were then segmented and manually labelled by one researcher according to the observed behaviour in the synchronised video footage using a custom MATLAB graphical user interface (GUI) designed for behavioural annotation (MATLAB v23.2.0, R2023b, [41]). Sixteen behaviours were initially identified and described: sleeping/resting, tree sitting, foraging, grooming, body shake, climbing up, climbing down, rapid climbing up, branch walking, swinging/hanging, general/unclassified in-tree movement, bellowing, walking, trotting, galloping, and bounding on the ground (Table S1). Some behaviours were under-represented in the training data and were either grouped with related behaviours or clustered in final model predictions and post-processing analyses.

### 2.6. Building the Training Dataset

The final training dataset used 11.02 h of labelled accelerometry data across 6 axes from the two captive koalas (recorded at 100 Hz, but down-sampled to 50 Hz), and the best samples of in-field behaviours collected from the two free-roaming koalas (recorded at 50 Hz). Behaviours with sufficient samples (i.e., Walking, Feeding, Grooming) were retained as distinct categories, while others with limited samples or biomechanical similarities were grouped (i.e., Still: Sleeping and Tree Sitting; In-tree Movement: Branch Walking, Swinging/Hanging, and all other undifferentiated in-tree movements; Climbing: Climbing Up, Climbing Down). Seven broader behavioural categories were used for classification: In-tree Movement, Still, Feeding, Grooming, Walking, Climbing, and Bellowing.

All data cleaning and analyses were conducted in R (v4.5.0; R Core Team, Vienna, Austria, 2024). The dataset was segmented into 1 s windows with 50% overlap between windows, meaning that each new segment shares half of its data with the preceding window, allowing for smoother temporal transitions between segments. This overlap helps to capture patterns that might span across window boundaries, ensures the model is more sensitive to subtle or transitional behaviours, and acts as a form of oversampling to increase data size [53]. The statistical features used were drawn from Tatler et al. (2018) and all time series features from the R package tsfeatures ([54,55]; see archived code for full list).

As this model was trained on captive individuals but deployed on different free-roaming individuals, the optimal validation strategy for this model would have been to stratify by individual following a Leave-One-Individual-Out strategy [56,57]. However, as full behavioural profile data were collected from only two individuals, this was not possible. The next most appropriate testing protocol was to use a chronological time stratification process [56,58]. A chronological split ensures that while data from the same individual appear in the training and testing sets, no continuous behavioural sequences appear in both sets, decreasing the chances of overfitting and performance inflation [56]. Data were split chronologically for each behaviour per individual into sections for training (first 60%), validation (middle 20%), and testing (remaining 20%). Once estimates had been obtained, all data were used for the final model training.

### 2.7. Model Tuning and Training

Hyperparameters are variables of the machine learning model that cannot be learnt from the data but must be set prior to training. In this case, a Random Forest model architecture was used, with free hyperparameters (number_trees, mtry, and max.depth). Hyperparameters were tuned using the R package rBayesianOptimisation (Table S2). The Bayesian search was optimised for the optimal macro-average F1 score (weighted by class prevalence). The model with the highest overall F1 score on the validation dataset was selected as the optimal model (Table S2 for optimal model variables). The performance of this model was then assessed on the test data to obtain a final confusion matrix, with performance metrics represented in Table 1 (see Figure S1 for visualisation of the confusion matrix containing all predicted behaviours). The final model was used to make predictions on the unlabelled feature data from all wild koalas, which assigned behaviour predictions to each 1 s window.

### 2.8. Post-Processing

The final output for each koala included a predicted behaviour class and the maximum and minimum Vectorial Dynamic Body Acceleration (VeDBA) calculated for every second of the study period. VeDBA reflects the sum of dynamic body acceleration across all three axes and is widely used as a proxy for energy expenditure, as it correlates with metabolic rate across a range of taxa [43,59,60]. Higher VeDBA values indicate greater movement intensity and metabolic costs.

Behavioural categories were grouped during post-processing: Feeding and Grooming were combined; Climbing was reclassified as *General Movement in Tree*; and Bellowing, Bounding, Rapid Climbing Up, and Body Shake were grouped as *Other*. Because the *Other* category represented < 0.1% of the data, it was excluded from subsequent modelling and summaries. The resulting four behavioural categories were: *General Movement in Tree*, *Motionless in Tree*, *Feeding & Grooming in Tree*, and *Walking*. Here, *Walking* refers exclusively to ground-based walking events and does not include walking along branches in trees or other behaviours occurring on the ground.

To reduce second-to-second noise without losing true short bouts, we first smoothed the 1 s behaviour labels with a centred 5 s modal filter (window ± 2 s). In each 5 s window, we took the most common label and reassigned the middle second to that label; in cases of ties, the original central label was retained. We then removed any ‘blips’ by collapsing runs < 3 s to the surrounding state and filling any gaps using last/next observation carried forward (zoo; [61,62]). Next, we split the smoothed 1 s series into non-overlapping 20 s bins and assigned each bin the majority label across its 20 samples. For each 20 s bin, we summarised VeDBA using the mean of the within-window maxima (VeDBA_max_) and minima (VeDBA_min_). We used this peak-sensitive summary to capture brief, high-intensity bursts (e.g., brief terrestrial locomotion) that simple means can occasionally wash out. The 20 s window length was chosen based on captive and field observations indicating that some behaviours occur within ~20 s—short enough to capture brief events while summarising longer bouts (e.g., resting). Sex, habitat type (patch vs. linear vegetation), and body mass were appended to each behaviour record.

Activity patterns over the 24 h cycle (periodicity in VeDBA_max_) were modelled using Generalised Additive Mixed Models (GAMM) with a cyclic cubic spline for hour of day and random intercepts for individuals (mgcv; [63]). Population-level variability was represented by the global SD of residuals around the fitted activity curve.

Differences in energy expenditure among behavioural categories were assessed using VeDBA_max_ as a proxy. Values were log-transformed to address right skew. We used a Kruskal–Wallis rank sum test for overall differences, followed by pairwise Wilcoxon rank sum tests with Benjamini–Hochberg correction for multiple comparisons.

Behavioural budgets were derived from direct counts of the non-overlapping 20 s bins (post-smooth). Counts were converted to seconds and aggregated by behaviour, individual, day, and hour of day to compute mean minutes per day, frequencies, and proportions over 24 h, and to identify peak times. To ensure comparability across days with recording gaps, daily totals were rescaled to 1440 min before computing percentages. Percentages were first averaged within individuals across days, then summarised across koalas as mean ± SD.

Because *Walking* was the primary behaviour of interest, we grouped consecutive *Walking* bins into Ground Visits. A Ground Visit was defined as a cluster of *Walking* bins separated by ≤60 s, with any gap greater than 60 s marking the start of a new visit. For each visit, we recorded the start time, end time, duration, number of visits per 24 h, and total time spent on the ground. Ground Visits represent only detected *Walking* and exclude other non-walking behaviours that may occur on the ground (e.g., sitting, grooming, scent-marking), providing a conservative estimate of visit duration but a consistent measure of visit frequency and timing.

Given the rarity of *Walking*, we fitted two GAMMs for proportions over the day. The first included all behaviours simultaneously with behaviour-specific cyclic smooths for the hour of day; however, *Walking*’s low prevalence limited power in the combined model. We therefore fitted a second GAMM restricted to *Walking*, with hour of day (cyclic smooth) and individual as a random effect, to detect its temporal pattern. Both models used REML, and we show predictions with ± SE ribbons. Finally, we used a linear mixed-effects model (lme4; [64]) to test for differences in time spent *Walking* between sexes and between habitat types (patch vs. linear vegetation), with individual ID as a random intercept to account for repeated measures.

## 3. Results

### 3.1. Activity-Related Energy Expenditure (VeDBA)

**Figure 2:**
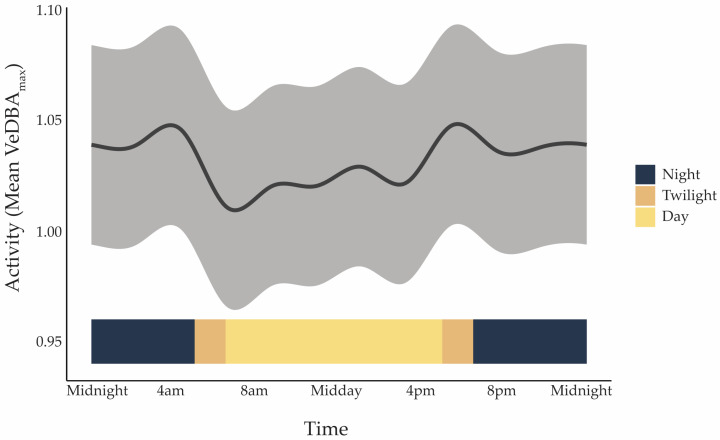
Mean activity level represented by Vectorial Dynamic Body Acceleration (VeDBA_max_; m·s^−2^) of koalas over a 24 h cycle, pooled across all individuals (*n* = 9), predicted from a Generalised Additive Mixed Model (GAMM). The grey shaded ribbon shows the model prediction with ±1 SD of the residuals. The coloured ribbon along the bottom indicates the timing of light phases in Pittsworth in May 2023: Night (18:30–05:00), Twilight (05:00–06:30 and 17:00–18:30), and Day (06:30–17:00). Peaks correspond to crepuscular periods, with reduced movement during daylight hours.

Koalas were most active at dawn, dusk, and into the early evening, with the highest level of activity, represented by Vectorial Dynamic Body Acceleration (VeDBA_max_), occurring between 17:00 and 18:00 (Figure 2). All individuals showed the same daily nonlinear cyclic rhythm (effective degrees of freedom [edf] = 9.99, F = 2597, *p* < 0.001), though some were more active overall than others (edf = 7.99, F = 1980, *p* < 0.001). On average, baseline VeDBA_max_ was 1.03 ± 0.0035 m·s^−2^. The statistical model confirmed strong daily cycles and individual differences but explained only 9.5% of the variation in activity (adjusted R^2^ = 0.095), suggesting that other factors are also important. Sex, habitat type (linear vs. patch), and body mass did not significantly influence activity in this dataset.

**Figure 3:**
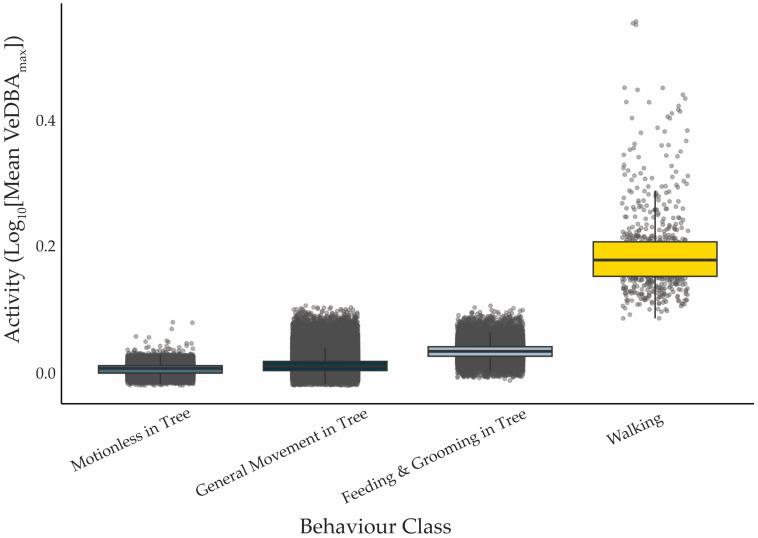
Mean activity level (VeDBA_max_; m·s^−2^) associated with each behavioural category, pooled across all koalas (*n* = 9) and days. Solid black lines indicate the median, boxes represent the interquartile range, error bars represent 1.5 × IQR, and grey points show the data points. All behavioural categories differ significantly from one another (*p* < 0.001).

Activity (VeDBA_max_) differed significantly among behavioural categories (χ^2^ = 99,859, df = 3, *p* < 0.001). *Walking* was associated with the highest VeDBA_max_ values, indicating greater exertion compared to other behaviours (all pairwise comparisons *p* < 0.001; Figure 3). *Feeding & Grooming in Tree* showed intermediate energy costs, while koalas spent a substantial portion of the day either moving or motionless in trees with relatively low body acceleration (Figure 3, Table 2).

### 3.2. Daily Patterns of Behaviour in Trees

In a 24 h period, koalas spent most of their time moving in trees (827.3 ± 204.7 min per day), followed by periods of rest or motionlessness in trees (385.2 ± 194.3 min per day). Feeding and grooming in trees accounted for an average of 224.2 ± 39.2 min daily. Koalas showed clear daily patterns across all tree-based behaviours, with significant nonlinear cyclic variation over the 24 h period (edf*_Feeding&Grooming_* = 7.00, F = 17.434, *p* < 0.001; edf*_GeneralMovement_* = 5.89, F = 6.14, *p* < 0.001; edf*_Motionless_* = 4.56, F = 4.56, *p* < 0.001). The timing of behaviour peaks varied: motionlessness was most frequent between 07:00 and 08:00, movement in trees increased around 13:00 to 14:00, and feeding/grooming peaked in the early evening between 18:00 and 19:00 (Table 2). Individual koalas differed significantly in the proportion of time spent in each behaviour (edf = 6.67, F = 4.67, *p* < 0.001). The model explained 26.3% of the deviance (adjusted R^2^ = 0.259). Sex, habitat type, and body mass did not significantly influence these patterns and were excluded from the final model.

### 3.3. Daily Patterns of Walking

**Figure 4:**
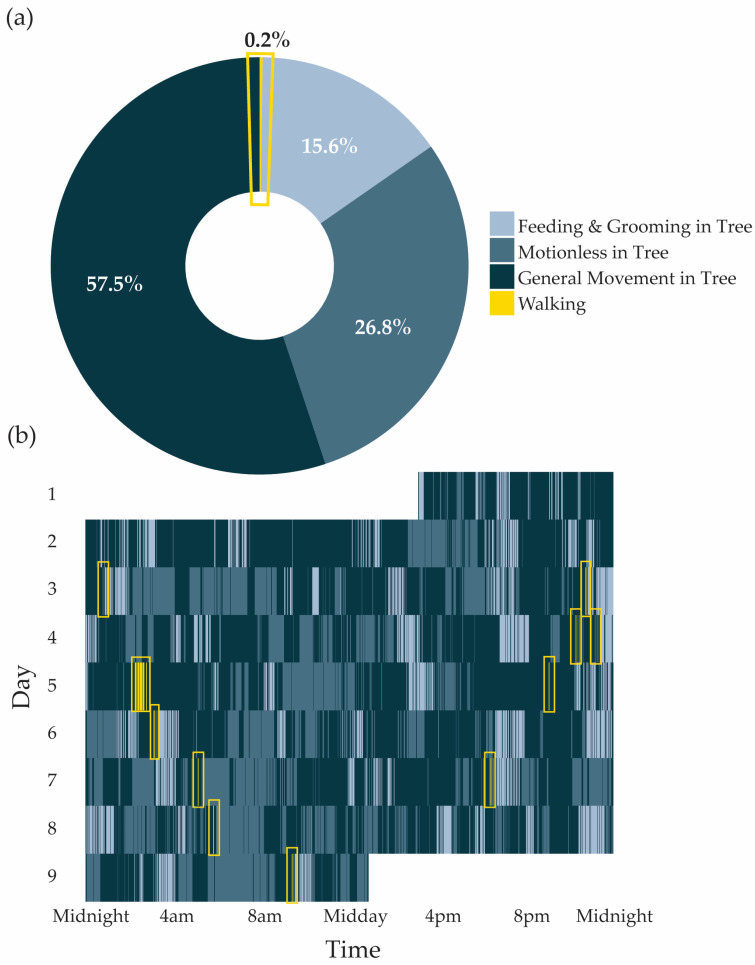
(**a**) The average proportion of time spent in each behavioural category across all koalas (*n* = 9) over a 24 h period. (**b**) Continuous daily behaviour sequence for one individual, “Hardy”, throughout the study period, with each tile representing a 20 s observation window coloured by behaviour. Ground Visits, identified by *Walking*, are highlighted by yellow boxes in both panels to emphasise their infrequent and brief occurrence within and across days.

**Figure 5:**
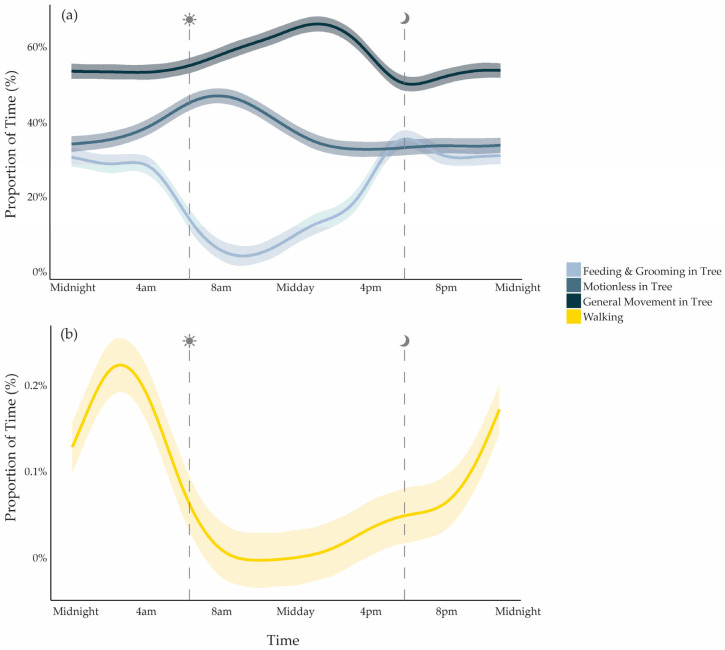
Modelled proportion of time spent in (**a**) arboreal behavioural categories and (**b**) *Walking* over a 24 h period, averaged across all koalas (*n* = 9). Lines show GAM-predicted values with shaded ribbons indicating 95% confidence intervals (CIs).

Koalas spent a small proportion of their day walking on the ground, averaging just 0.2% ± 0.1% per 24 h (3 ± 1.4 min; Table 2, Figure 4). Ground activity was most common in the early morning hours between 02:00 and 05:00 (Figure 5). On average, koalas went to the ground 2.95 ± 2.31 times per day, and each visit involved an average of 1.59 ± 1.95 min of walking. Because of the infrequency of *Walking*, we ran a separate GAMM to better capture temporal variation. This model confirmed strong daily nonlinear cycles in walking patterns, with clear peaks in nocturnal hours (edf = 4.78, F = 4.46, *p* < 0.001; Figure 5), but individuals did not differ in the time they spent walking (random effect of ID: *p* = 0.26). The model explained 20.3% of the variance in ground activity, suggesting that time of day was important, though clearly not the only factor shaping walking behaviour. Males did not differ from females in time spent *Walking* (F1,6 = 0.042, *p* = 0.84), nor did koalas differ between patch or linear habitats (F1,6 = 0.59, *p* = 0.47).

## 4. Discussion

Koalas (*P. cinereus*) are known to be largely sedentary, slow-moving, and seemingly predictable in their daily routines. Yet the most perilous part of a koala’s day—walking on the ground—has been largely overlooked by broad-scale tracking or direct observation alone [3,18]. Here, we present an early-stage yet powerful approach to studying the behaviour and walking patterns of koalas in the wild. Using high-frequency accelerometer data, we detected and quantified this infrequent behaviour, separating arboreal from terrestrial activity and identifying when, how often, and for how long koalas walked in a fragmented agricultural landscape. As predicted, walking was a transient but detectable behaviour, occurring almost exclusively at night and accounting for only 0.2% of all activity. Most of their time was spent resting, feeding, and moving within the canopy—behaviours that exhibited substantially lower activity levels than walking. Characterising this brief yet disproportionately risky period provides insight into a key source of mortality for koalas, as ground-based movement increases exposure to vehicles and dogs.

Beginning with an understanding of the temporal context in which koala behaviour occurs, koalas in our study showed clear nocturnality, with activity and overall energy expenditure peaking around dusk and dawn, consistent with earlier behavioural studies [20,22,34]. Arboreal behaviours dominated daily time budgets but followed distinct crepuscular temporal profiles: within-tree movement rose through the early afternoon as animals became active, feeding and grooming peaked overnight (with peaks in walking frequency often following peaks in feeding frequency), and long periods of rest occurred primarily during the day after nocturnal activity. Similar patterns have been reported in other studies, with peak activity in trees occurring between 6 pm and midnight [65], peaks in feeding activity around 7 pm and 5 am [22], and minimal time spent in active behaviours compared with prolonged rest [9,34]. By examining the energetic profiles of each classified behaviour, we found that arboreal behaviours produced lower dynamic body acceleration than walking, supporting our prediction that ground movement incurs higher movement-related costs than most within-canopy behaviours.

These temporal and energetic patterns reflect two linked aspects of koala ecology: specialised folivory and thermoregulation. First, a eucalypt diet is low in nutrients and high in fibre, tannins, and toxic compounds such as formylated phloroglucinol compounds (FPCs), requiring long periods of inactivity for digestion—seen as extended motionlessness in our dataset [7,66,67]. Second, koalas select cooler microclimates, denser canopies, and non-fodder trees with large trunks during warmer hours to reduce heat load and facilitate thermoregulation [3,22,68,69,70]. Concentrating higher-energy movement into cooler hours, therefore, likely balances thermoregulatory demands with foraging and locomotion costs. Although accelerometers cannot directly capture the metabolic costs of digestion—probably a major component of daily energy expenditure—the high movement costs of walking help explain why it, like feeding, concentrates in cooler hours, making it a rare but necessary exception to an otherwise low-movement day [18].

Walking was infrequent and tightly timed within this window of nocturnal activity. Koalas descended to the ground two to three times per night for only a few minutes per visit, with 2–5 am being the most active walking period. Earlier studies report a similar frequency and timing of nocturnal and crepuscular ground activity, with movement and road-crossing rates peaking at dawn and dusk [8,20,24,27,28,34]. However, our pre-dawn emphasis could reflect population-, landscape-, or season-specific differences, as our dataset was limited to nine individuals tracked for eight days during the non-breeding season in a single fragmented agricultural habitat. Further, habitat availability, predation risk, and access to thermal refuges differ markedly across landscapes, which may alter when and how often individuals descend trees. The strong temporal coupling between feeding and walking in our data suggests that most ground movement was driven by foraging requirements, although bellowing activity during the breeding season (peaking between midnight and 5 am [26]), indicates that acoustic signalling and territorial behaviour may also influence ground-use patterns, particularly among males [8,71].

We estimated that koalas spend 0.2% of their time walking, whereas previous studies report koalas spending 0.7–10% of their total time on the ground [20,24,27,28]. This difference likely reflects the model being optimised to detect walking, meaning locomotive bouts, specifically, were captured, but other ground behaviours—such as pausing to assess nearby trees, scent-marking, or resting between climbs—were not resolved and often absorbed into broader arboreal categories [8]. A similar mechanism may explain the discrepancy between our resting estimates (26.8%) and those reported in other behavioural studies (~70–80%; [34,72,73]): the classifier was trained on captive individuals in static trees and therefore did not incorporate low-magnitude canopy motion caused by wind, possibly causing sleeping koalas in swaying branches to be misclassified as active. Quantifying environmental noise is essential for future studies employing this method.

Utilising accelerometer data with machine learning can be a powerful method to classify behaviours across large datasets, but model validation remains a key challenge. Our model performed well within training individuals (macro F1 ≈ 0.76) but declined markedly when applied to new wild koalas (macro F1 ≈ 0.46), reflecting constraints of the training dataset. The model was trained on only two captive individuals observed in enclosures that lacked the structural complexity, canopy motion, and behavioural repertoire available to free-roaming animals, yet was subsequently applied to wild individuals in far more variable natural environments [55,74,75,76]. As a result, behaviours requiring large trees or ground space—such as extended climbing or bounding—were under-represented, and categories with similar biomechanics (e.g., feeding vs. low-intensity movement) were more frequently confused (Supplementary Figure S1, Table S3, [77]). These validation constraints mean our behavioural classifications—particularly arboreal behaviours—should be interpreted with caution. Therefore, this study represents an important first step in applying high-resolution accelerometry to free-ranging koalas, but broader validation using larger datasets across conditions and landscapes will be essential for refining behaviour-specific classifications. While we cannot definitively validate the classification accuracy for all behaviours in the wild data, the broad activity states (active vs. inactive), the timing of daily activity, and the detection of ground-based walking events aligned closely with patterns reported from GPS telemetry, direct observation, and acoustic monitoring [8,22,34].

Identifying when koalas walk provides insight into a comparatively understudied aspect of risk; however, interpreting the consequences of these movements requires understanding where they occur within different landscapes. Walking is necessary for accessing new feeding trees, mates, and resting sites, but in fragmented landscapes, most ground visits carry heightened risk. Koalas residing in urban and agricultural landscapes tend to have larger home ranges, travel further along more direct paths, and walk across roads more frequently than those in intact forests, often moving along roadside vegetation during dispersal [27,28,78]. Reduced habitat connectivity funnels individuals into small, isolated patches, making movements between trees longer, riskier, and more energetically costly [18]. Our study provides high-resolution baseline temporal data for one fragmented agricultural landscape, but implications that are applicable across koala populations more broadly will require comparisons of movement patterns across different landscape types, sexes, and seasons. Further, integrating fine-scale accelerometry with GPS tracking, remote cameras, thermal-imaging drones, habitat mapping, and physiological or acoustic sensors will be essential for determining how landscape structure, resource availability, and social context shape these rare but critical movements [37,38,76,79]. Complementary analyses of habitat use and spatial movement (forthcoming) will help place these temporal patterns within the broader context of landscape-level behaviour.

As terrestrial movement tends to be rare and temporally clustered, management actions may be most effective when aligned with periods of heightened ground activity. The narrow nocturnal windows when koalas are most active on the ground—particularly at dusk and pre-dawn—present opportunities for targeted intervention [20,80]. Speed reductions during peak movement hours can reduce wildlife–vehicle collisions [81,82,83], and signs help to alert drivers to periods and zones of heightened risk [80,84,85,86]. Wildlife crossings and exclusion fencing can be highly effective when strategically placed along known movement routes [13,83,87,88,89], but long-term success will also depend on addressing habitat fragmentation itself. Ultimately, reconnecting fragmented landscapes through habitat corridors and retaining scattered paddock trees and patches as stepping-stones reduces the need for risky ground movements while supporting functional connectivity across the landscape [2,14,30,90,91,92,93,94,95,96,97]. These approaches—from tactical temporal interventions to strategic habitat restoration—could benefit not only koalas but the broader suite of arboreal species facing similar threats in fragmented landscapes [40,98,99,100,101].

## 5. Conclusions

Using high-frequency accelerometer data and machine learning, we quantified a short-lived but critical behaviour in koalas in a fragmented agricultural landscape: ground movement accounts for only 3 minutes daily yet drives two-thirds of mortality. This temporal concentration of behaviour highlights potential opportunities for targeted conservation action, but comparisons across landscapes, seasons, sex, and age will be critical for determining their broader applicability. Beyond koalas, this approach illustrates how high-resolution behavioural data can reveal when and where cryptic species are most vulnerable. As habitat fragmentation, urbanisation, and climate change intensify pressure on wildlife, integrating accelerometry with spatial tracking and habitat data will be essential for matching conservation interventions to animal behaviour.

## Figures and Tables

**Table 1:** Performance metrics of the optimal Random Forest model used for classifying koala behaviours from accelerometer data.

Behaviour	Precision	Recall	F1	Accuracy	Prevalence
Class: Bellowing	0.791667	0.142857	0.242038	0.571197	133
Class: Climbing	0.780275	0.788146	0.784191	0.885394	793
Class: Feeding	0.762519	0.834025	0.796671	0.900923	1205
Class: Grooming	0.734861	0.683409	0.708202	0.824861	1314
Class: Still	0.998971	0.95334	0.975622	0.976558	2036
Class: In-tree Movement	0.765428	0.837678	0.799925	0.880148	2532
Class: Walking	0.991938	0.96951	0.980596	0.98332	2919
Macro-average	0.832237	0.744138	0.755321	0.860343	NA

Precision reflects the proportion of predicted positive instances that are correct, recall indicates the proportion of actual positive instances that are correctly identified, and the F1 score is the harmonic mean of precision and recall. Accuracy represents the overall proportion of correctly classified instances. Prevalence indicates the number of instances per class in the test dataset. The macro-average provides an unweighted mean of these metrics across all categories, summarising overall model performance, and therefore does not have an associated prevalence score.

**Table 2:** Summary statistics for behavioural categories of wild koalas (*n* = 9) as predicted by the best-performing Random Forest model.

Behaviour	Minutes per Day (Mean ± SD)	Percent of Day (Mean ± SD)	Peak Occurrence (24 h)	Frequency per Day (Mean GV ± SD)	Duration per GV (Mean Minutes ± SD)
Motionless in Tree	385.2 ± 194.3	26.8% ± 13.5%	07:00–08:00	-	-
General Movement in Tree	827.3 ± 204.7	57.5% ± 14.2%	13:00–14:00	-	-
Feeding and Grooming in Tree	224.2 ± 39.2	15.6% ± 2.7%	18:00–19:00	-	-
Walking	3 ± 1.4	0.2% ± 0.1%	02:00–03:00	2.95 ± 2.31	1.59 ± 1.95
Other	0.2 ± 0.5	-	-	-	-

Values represent the mean ± SD of minutes per day and percentage of time spent in each behaviour, calculated by first averaging daily occurrences within each individual and then across all individuals. Also shown are the typical daily peak time (24 h format), and the frequency and duration of ground visits per day, estimated from grouped windows of *Walking*. Ground Visit (GV) was defined as a cluster of *Walking* 20 s windows, with gaps greater than 60 s delimiting separate visits. For each visit, start time, end time, and duration were recorded. Percentages were scaled so that each day sums to 100%. The ‘Other’ category represented < 0.1% of the data and is included for completeness only.

## Data Availability

The datasets presented in this study are not readily available because the data are part of an ongoing study. Requests to access the datasets should be directed to the corresponding author(s). All code and supporting materials for analysis and the publicly available GitHub (v3.19.0) can be found at: https://github.com/OakAlice/KoalaAnalysis, accessed on 2 December 2025.

## Supplementary Materials

The following supporting information can be downloaded at: https://www.mdpi.com/article/10.3390/ani15243537/s1, Table S1: Definitions and accelerometer examples describing the 16 behavioural classes identified in the original training dataset prior to grouping. Table S2: Bounds used in the model tuning process. Table S3: Variables identified in the optimal model from the tuning process using rBayesianOptimisation. Figure S1: Visualisation of the confusion matrix and performance of the model on the test data containing all initially identified/described behaviours.

## Abbreviations

The following abbreviations are used in this manuscript:

VeDBA	Vectorial Dynamic Body Acceleration
GAMM	Generalised Additive Mixed Model
edf	Effective degrees of freedom
