# Supplementary material for: Between the Trees: Quantifying Koala Ground Movement for Conservation Action
Source: Animals (Basel). 2025 Dec 8;15(24):3537. doi: 10.3390/ani15243537 (PMC12729488; doi:10.3390/ani15243537)
Supplement: Supplementary file 1 [file animals-15-03537-s001.zip › animals-4015645-supplementary.pdf]

Supplementary Materials

Between the Trees: Quantifying Koala Ground Movement for Conservation Action

Gabriella R. Sparkes, Oakleigh Wilson, William A. Ellis, Sean I. FitzGibbon, Benjamin J. Barth, Christofer J. Clemente, Mathew S. Crowther, and Robbie S. Wilson

Table S1: Definitions and accelerometer examples describing the 16 behavioural classes identified in the original training dataset prior to grouping.

Original Behaviour	Definition	Accel Signature Example	Final Grouping
Sleeping/ resting	Koala is motionless, eyes closed or half-closed, limbs supported by trunk or branch, typically curled or slumped posture.	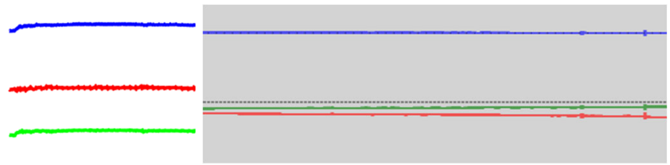	*Motionless in Tree*
Tree sitting	Koala is upright or semi-upright, awake but inactive, alert to surroundings and looking around, but not feeding or moving.	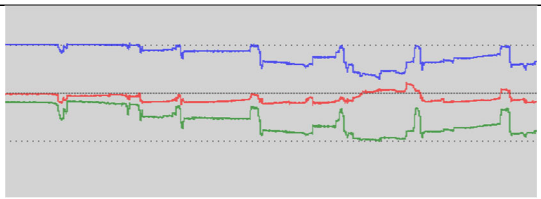	*Motionless in Tree*
Foraging	Reaching for leaves, plucking, chewing, repeated small forelimb movements, head and neck movement while browsing.	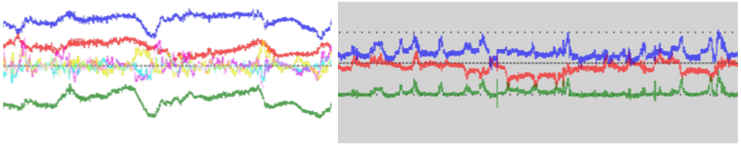	*Feeding and Grooming*
Grooming	Scratching or rubbing with hind- or fore-foot. Similar signatures to foraging but on shorter timescales.	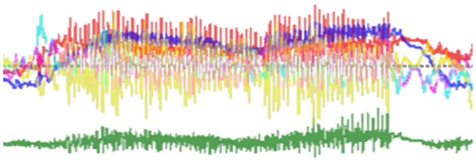	*Feeding and Grooming*
Body shake	Rapid, full-body or just head shake. Maxes out the acceleration signature.	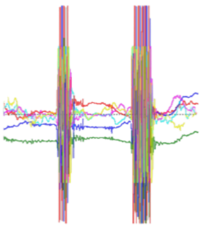	*Other*

Climbing up	Ascending a tree trunk or vertical branch using alternating forelimb-hindlimb cycles.	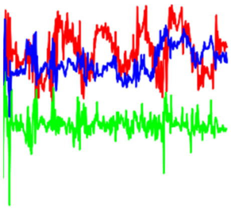	*General Movement in Tree*
Climbing down	Descending a tree trunk or vertical branch using alternating forelimb-hindlimb cycles, often more cautiously, with slower cycles and more noise around the trace compared to Climbing Up.	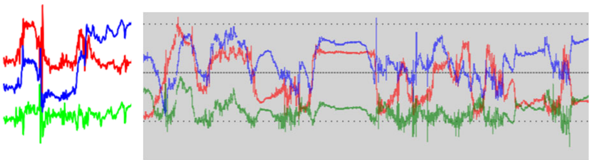	*General Movement in Tree*
Rapid climbing up	Fast ascent up a tree trunk or vertical branch, similar biomechanics to bounding, usually in response to disturbance or at release in the wild. Example on the right shows the end of a ground walk, transitioning into a rapid climb up (outlined by the black box), followed by a regular climb up.	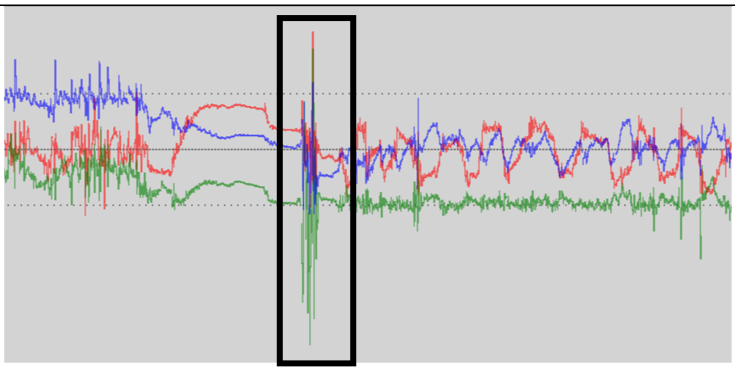	*Other*
Branch walking	Moving laterally along a branch to relocate within the canopy, deliberate stepping along a branch, similar biomechanics to walking but with more sway/noise in the signal.	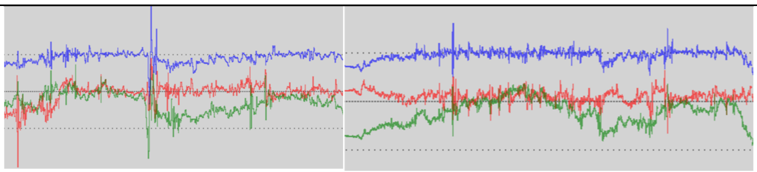	*General Movement in Tree*
Swinging/ hanging	Supporting body weight on forelimbs or hindlimbs, shifting body while suspended or moving between branches. Typically, multiple movements at once (produces very messy trace).	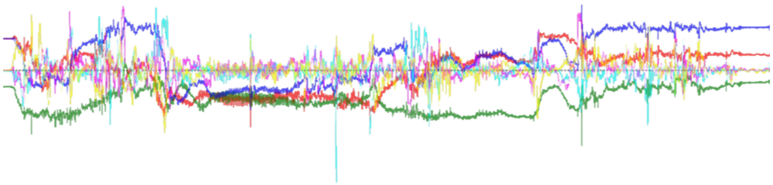	*General Movement in Tree*
General/unclassified in-tree movement	Mixed/vague movements within the canopy that do not clearly match a defined behaviour—minor repositioning, short locomotor transitions, brief directional changes. Often involves other arboreal behaviours.	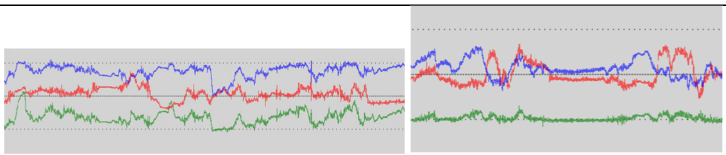	*General Movement in Tree*

Bellowing	Deep, resonant male vocalisation, mix of long droning sounds and rhythmic pulsing sounds, usually with head angled up and backwards, tense body, and can reverberate through the body.	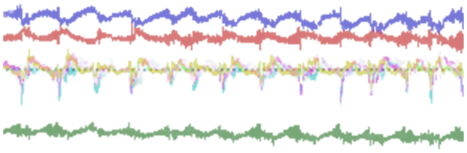	*Other*
Walking	Four-limb quadrupedal movement on the ground at a moderate pace, typical locomotor gait during ground travel.	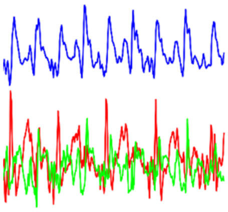	*Walking*
Trotting	Faster quadrupedal ground movement, limbs move more in diagonal pairs.	NA – Insufficient samples	NA
Galloping	High-speed ground locomotion, forelimbs and hindlimbs moving in grouped sequences.	NA – Insufficient samples	NA
Bounding	Rapid, leaping ground movement, typically used in escape or urgent travel, often on release in the wild, both hindlimbs may push off simultaneously.	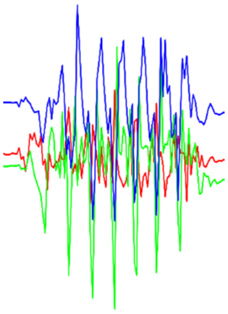	*Other*

Table S2: Bounds used in the Bayesian Optimisation model tuning process.

**Category**	**Variables Tested**
Mtry	2 – 50
Max_depth	5 – 30
Number_trees	100 – 1000

Table S3: Variables identified in the optimal model from the tuning process using rBayesianOptimisation.

**Category**	**Optimal Variables**
Mtry	9.4484
Max_depth	24.2455
Number_trees	169.7472
Macro F1	0.6951

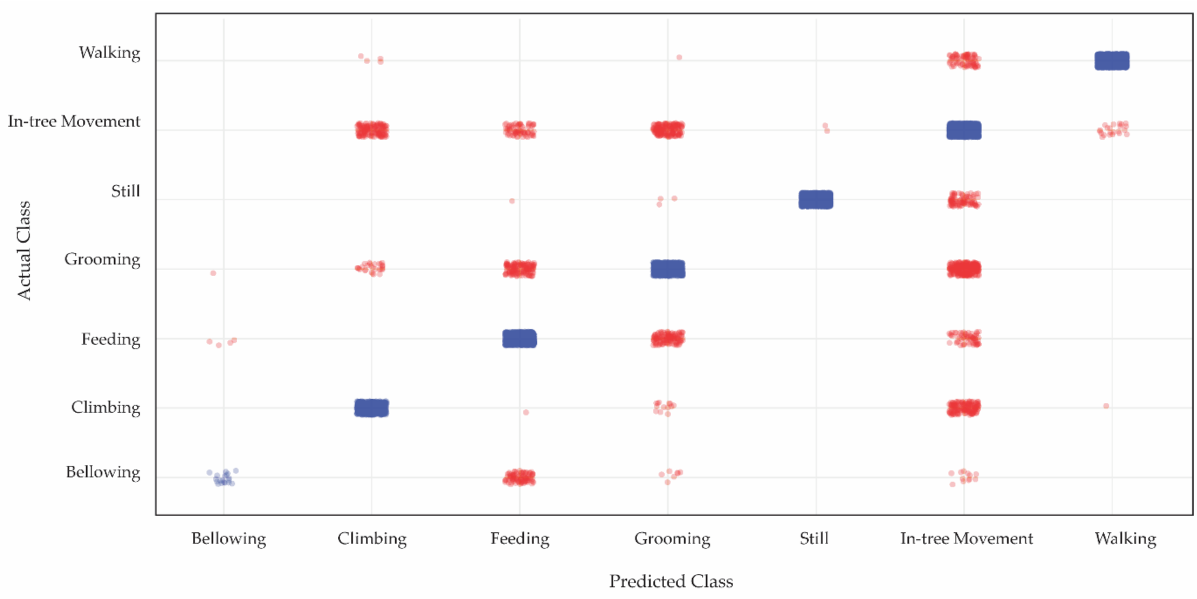

Figure S1: Visualisation of the confusion matrix and performance of the model on the test data containing all initially identified/described behaviours.
